# Neurohumoral, immunoinflammatory and cardiovascular profile of patients with severe tetanus: a prospective study

**DOI:** 10.1186/1477-5751-5-2

**Published:** 2006-02-17

**Authors:** Janete S Brauner, Nadine Clausell

**Affiliations:** 1 Intensive Care Units from Hospital Nossa Senhora da Conceição and Hospital de Clínicas de Porto Alegre, Rua Ramiro Barcelos, 2350/2060, CEP 90035-003, Porto Alegre, RS, Brazil. Graduate Program in Cardiology and Cardiovascular Sciences, Universidade Federal do Rio Grande do Sul, Porto Alegre, RS, Brazil; 2 Graduate Program in Cardiology and Cardiovascular Sciences, Universidade Federal do Rio Grande do Sul, Porto Alegre, RS, Brazil

## Abstract

**Introduction:**

Autonomic disturbances in tetanus are traditionally associated with adrenergic variations and/or cardiac dysfunction, based on case report data. The objective of this study was to measure catecholamines, (TNF)-α and troponin T relative to and left ventricular ejection fraction (LVEF) in patients with severe tetanus.

**Methods:**

This prospective study was carried out at two general Intensive Care Units and included 21 patients consecutively admitted with severe tetanus. Catecholamines (dopamine, norepinephrine, epinephrine and total catecholamines), tumor necrosis factor (TNF)-α and LVEF were assessed during the first week of autonomic instability and following tetanus recovery. Troponin T was measured during autonomic instability only.

**Results:**

Mean age of patients was 46 ± 17 years, median Acute Physiology and Chronic Health Evaluation II (APACHE II) score was 8 (range 1–23). All patients had both blood pressure and heart rate instability. Two patients were recuperated from cardiac arrest. Intensive Care Unit mortality was 14% (3 cases). No increase in total catecholamines or in TNF-α levels was observed during autonomic instability or in the recovery period. Six patients had troponin T >0.01 ng/ml and six had >0.1 ng/ml. Mean LVEF was similar during autonomic instability and after tetanus recovery, 67 ± 7% and 65 ± 7%, respectively. Troponin T levels correlated with pressoric instability during autonomic instability.

**Conclusion:**

Our study demonstrated that in patients with severe tetanus no significant increased levels of catecholamines or TNF-α or evidence of cardiac systolic dysfunction was observed either during autonomic instability or in the recovery period. Elevated values of troponin T detected during autonomic instability were not associated with left ventricular dysfunction. Our data do not support the hypothesis that autonomic disturbances in tetanus are associated with adrenergic variations or cardiac dysfunction.

## Introduction

Autonomic dysfunction, sudden death and complications of prolonged critical disease, such as nosocomial infections, thromboembolism and gastrointestinal bleeding are main causes of death in tetanus [1]. In a previous study we showed that autonomic instability occurred in 100% of patients with tetanus, and that 35% of deaths were timely associated with the symptomatic period, cardiac arrest and hypotension, and inversely associated with the duration of the onset period [2].

Autonomic instability as defined by Kerr et al., is a characteristic syndrome whose features include sustained but labile hypertension and tachycardia, irregularities of cardiac rhythm, peripheral vascular constriction, profuse sweating, pyrexia, increased carbon dioxide output, increased urinary catecholamine excretion, and, in some cases, the development of hypotension [3]. Severe hypertension and tachycardia may alternate with profound hypotension and bradycardia, suggesting intense sympathetic activity [3,4]. Accordingly, both increased urinary and plasmatic levels of catecholamines have been described in this setting [3,5]. Other authors, however, found normal excretion and plasma catecholamine levels in patients with tetanus [5,6], indicating that an association between elevated catecholamine levels and autonomic instability remains to be further documented. Bradycardia is also occasionally noticed, which suggests parasympathetic system or basal nucleus dysfunction, leading to excessive vagal activity [7,8].

Prolonged stimulation of the sympathetic nervous system or the continuous release of catecholamines may cause vascular and myocardial damage [9,10]. However, histologic evidence of myocardial necrosis in tetanus was demonstrated in few cases [11]. It was suggested that either a sudden loss of catecholamine stimulation or myocardial damage caused by the direct action of the tetanus toxin, could be involved in cardiac dysfunction described in tetanus [12,13]. However, an invasive hemodynamic study involving 27 patients with severe tetanus showed a hyperdynamic profile rather than depressed cardiac function [14]. Since it is well known that myocardial damage caused by catecholamines can induce synthesis of cytokines by myocytes [15,16], cytokines, specifically those with known cardiodepressant properties such as TNF-α, could be an alternative mechanism involved in cardiac dysfunction, in the setting of tetanus.

The objective of the present study was to evaluate the temporal behavior of catecholamines and of TNF-α relative to left ventricular ejection fraction obtained by two-dimensional transthoracic echocardiography at two time points: during autonomic instability and after recovery from tetanus. Levels of the troponin T were used to identify potential myocardial damage in the period of autonomic instability. In this report our data failed to indicate associations between adrenergic and cytokine activation with changes in cardiac performance in patients with tetanus.

## Methods

### Study population

This cohort study included all patients with a diagnosis of severe tetanus characterized by marked rigidity, frequent generalized spasms, dysphagia, respiratory compromise or apnea according to the modified Abblett's scale, [17,18] consecutively admitted to the ICU of two general hospitals (Hospital Nossa Senhora da Conceição and Hospital de Clínicas de Porto Alegre, Brazil). Data were prospectively collected without interference in management. Patients with other potential causes of hemodynamic instability such as septic shock were excluded from the study. The study was approved by the Ethics Committees from both hospitals. Patients' legal representatives signed an informed consent document prior to enrolment. A portion of this population was part of the entire cohort of our previous study reporting demographics and prognosis of patients with tetanus [2].

The main variables of the study were: plasma catecholamines, TNF-α, troponin T levels and transthoracic echocardiographic-based LVEF. These variables were assessed both during the autonomic instability and after recovery from tetanus, with the exception of troponin T, only measured during autonomic instability. The following variables were also recorded: age, sex, APACHE II score in the first 24 hrs of ICU admission, temporal development of symptoms, periods of the disease (incubation period, onset period, symptomatic period), clinical characteristics, clinical and infectious complications and electrocardiogram (EKG).

### Evaluation of autonomic profile, catecholamine levels and TNF-α

Autonomic instability was characterized by the presence of blood pressure and heart rate lability, arrhythmias and/or cardio respiratory arrest recorded by continuous noninvasive monitoring (monitor 66S, Hewlett-Packard, USA), as reported previously [2]. Variation from minimum to maximum (delta) of blood pressure and of heart rate was recorded in both periods.

At the end of the first week of autonomic instability and after recovery from tetanus, 10 ml blood sample was collected into an EDTA-containing tube and kept under refrigeration. Samples were immediately centrifuged at 5°C, plasma was placed in 1.5 ml Eppendorf tubes and stored at -80°C for later measurement of plasma catecholamines and TNF-α levels. Catecholamine levels (epinephrine, norepinephrine, dopamine and total catecholamines) were measured at CRIESP laboratory (São Paulo, Brazil) using high performance liquid chromatography (HPLC). Commercially available Elisa assays were used to measure TNF-α plasma levels using duplicate samples to minimize inter-assay variability. Lower detection limits of the assay were typically less than 4.4 pg/ml (R & D Systems, Minneapolis, MN, USA).

### Troponin T levels during autonomic instability

During autonomic instability, blood samples collected were also used for measurement of plasma troponin T levels using a sandwich electrochemiluminescence immunoassay (ECLIA), Elecsys Troponin T STAT (Roche, Germany). Detection band ranges from 0.01 ng/ml to 25 ng/ml in this assay. In 99% of healthy volunteers, the cutoff point was lower than 0.01 ng/ml, and the cutoff point for myocardial infarction was 0.1 ng/ml [19-21].

### Transthoracic echocardiography functional evaluation

Two-dimensional color Doppler transthoracic echocardiography was performed at the two collection time points (autonomic instability and after recovery period) to measure LVEF by the M-mode (Teicholtz method), in accordance to the recommendations of the American Society of Echocardiography. Whenever possible, hemodynamic status of patients were kept at the best possible care according to the ICU protocols in order to avoid confounding load variables interfering with ejection fraction measurements. Simultaneously to echocardiogram, blood pressure and heart rate were recorded as well as blood sampling to measure biological variables. Additional echocardiography-based cardiac findings were also recorded. Data were recorded and later re-evaluated by another blinded echocardiographist.

### Statistical analysis

Continuous variables are described as means and standard deviation or medians and range; categorical variables are described by frequency tables and proportions. Magnitude of variation from minimum to maximum values of variables (heart rate and blood pressure) were calculated and expressed as delta values. Discrimination between parametric and non-parametric variables was performed using histograms and the Kolmogorov-Smirnov test. Student *t *test was used to compare continuous and normally distributed variables; Mann Whitney test was used for asymmetric continuous variables, and the chi-square test for categorical variables. Paired samples *t *tests and Wilcoxon test were used to compare continuous variables during and after autonomic instability. Pearson and Spearman tests were used to evaluate correlation between variables. Significance level was established at p < 0.05 for all comparisons.

## Results

### Clinical characteristics

We evaluated 21 patients with severe tetanus, 18 (84%) males, mean age 46 ± 17 years, with median APACHE II score of 8 (range – 1–23). Incubation period was 7.0 ± 4 days, onset period was 3 ± 3 days and symptomatic period was 40 ± 10 days. All patients were mechanically ventilated (mean 41 ± 12 days) and the length of stay in the ICU was 45 ± 13 days. ICU mortality rate was 14% (three patients), thus 18 patients underwent after recovery assessments. Seven patients had chronic obstructive pulmonary disease, six had history of alcohol abuse, three had hypertension, and three had history of coronary artery disease. Patients received diazepam (mean dose = 33.9 ± 8.0 mg/h), pancuronium (mean dose = 0.8 ± 0.08 mg/kg/h) and morphine (median dose = 3 mg/h; range – 0–20) intravenously, continuously. In addition to drugs used for sedation, seven patients also received clonidine 0.150 mg/day. All patients received antibiotics – nine for tetanus only, and 12 for other infections also. The most frequent clinical and infectious complications were: pulmonary atelectasis (57%), renal failure (24%), respiratory infection (90%), urinary infection (81%), and central line infection (38%). Individual clinical characteristics are shown in Table 1.

**Table 1:** Clinical and laboratory findings during autonomic instability at time of collection.

**Patient**	**Age**	**APACHE**	**MBP**	**HR**	**CREAT**	**TROP T**	**Infection**	**Germ**	**AB**
MAPRR	20	6	145	129	0.8	<0.01	Blood	*S. aureus*	vancomycin
VPO	48	3	110	127	1.1	0.012	-	-	penicillin
MSR	54	4	49	109	0.5	0.07	respiratory central line	*S. aureus*	vancomycin
JP	50	8	84	124	0.6	<0.01	respiratory	?	cefuroxime
JRS	74	10	78.6	122	1.4	0.306	-	-	penicillin
ESB	19	6	62	81	0.8	<0.01	-	-	penicillin
CEO	53	8	78.6	92	0.5	0.135	respiratory	*S. aureus*	vancomycin
RAB	57	6	69	74	1.1	0.015	-	-	penicillin
JOR	57	15	100.6	129	1.0	0.028	osteomyelitis	-	ofloxa, metro
JBS	48	10	90	98	0.7	0.02	-	-	penicillin
GS	36	11	67	96	1.0	0.045	respiratory	*S. aureus*	vancomycin
SBD	33	15	88	71	0.8	0.105	respiratory	?	cefipime
LCCM	48	4	93.6	62	0.6	<0.01	respiratory	*P. mirabilis*	ampisulbact
MJO	29	1	87	88	0.7	<0.01	-	-	penicillin
JCWV	20	5	290	133	0.7	<0.01	respiratory	?	ampisulbact
IMR	60	23	66	86	0.7	<0.01	respiratory	*P. aeruginosa*	ampisulbact
MS	70	11	48.3	78	1.1	0.137	Blood	*S. aureus*	vancomycin
ER	44	11	75	81	1.1	0.135	Blood	*S. epidermitis*	oxacillin
LSLF	36	10	70	100	1.0	<0.01	-	-	ampicillin
MLS	82	21	24	92	1.5	0.2135	-	-	penicillin
JS	44	2	126	75	1.0	<0.01	-	-	penicillin
Mean (SD)	46.7 (17.2)	9 (5.7)	81.3 (26.7)	97.4 (22.1)	0.9 (0.2)	0.6 (0.08)			

SD = standard deviation; MBP = mean blood pressure; HR = heart rate; CREAT = creatinine; TROP T = troponin T; AB = antibiotic (ofloxa = ofloxacin; ampisulbact = ampicillin/sulbactam; metro = metronidazole).

### Autonomic profile

All patients had autonomic instability characterized by blood pressure or heart rate variation, other arrhythmias or cardiac arrest. During autonomic dysfunction, mean maximum heart rate was 143 ± 17 bpm and mean minimum heart rate was 59 ± 18 bpm. Ten patients had marked bradycardia (two had third degree AV block requiring pacemaker implants), two had recuperated cardiac arrest and one had atrial fibrillation. Mean blood pressure during hypertension periods ranged from 109 to 199 mmHg and from 15 to 73 mmHg during hypotension periods.

### Catecholamine levels

**Figure 1:**
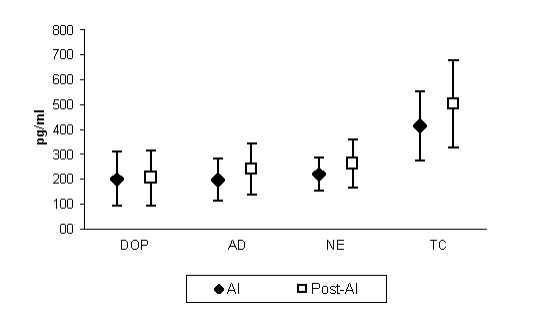
Levels of different catecholamines (pg/ml) at the two collection time points. Mean and standard deviation values. DOP = dopamine; AD = epinephrine; NE = norepinephrine; TC = total catecholamines; AI = autonomic instability; Post-AI = after recovery from tetanus.

Catecholamine concentrations were measured during the first week of autonomic instability and after recovery from tetanus. When plasma concentrations in the two periods were compared, we observed that levels of epinephrine (195 ± 83 *versus *239 ± 105 pg/ml), norepinephrine (218 ± 88 *versus *261 ± 96 pg/ml), dopamine (198 ± 109 *versus *204 ± 111 pg/ml) and total catecholamines (414 ± 138 *versus *500 ± 174 pg/ml) tended to be higher after recovery from tetanus, although within normal limits (Figure 1). Individual measurements showed levels above normal for dopamine in 11 patients, epinephrine in three patients, and norepinephrine in one patient during autonomic instability.

### TNF-α levels

**Figure 2:**
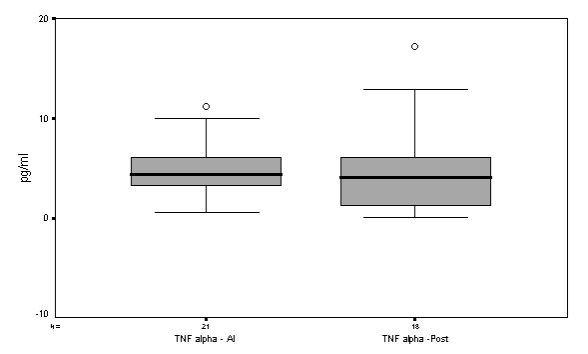
Box plot graph comparing TNF-α levels (pg/ml) at the two collection time points. Median and percentile values (25^th ^and 75^th^). AI = autonomic instability; Post-AI = after recovery from tetanus.

Levels of TNF-α showed similar median plasma concentrations in both periods, *i.e*. 4.5 (range -2.7–6.7) pg/ml and 4.1 (range -1.2–6.8) pg/ml in autonomic instability and after tetanus recovery, respectively p > 0.05 (Figure 2). Plasma levels of < 15.6 pg/ml were considered within normal expected values, according to the manufacturer.

### Troponin T concentrations and electrocardiogram

Analysis of plasma troponin T concentrations during autonomic instability revealed that nine patients had values lower than 0.01 ng/ml, and that 12 had values greater than 0.01 ng/ml. Of these, six patients had troponin T concentrations greater than 0.1 ng/ml. Patients with plasma troponin T concentrations greater than 0.01 ng/ml (n = 12) had greater delta systolic pressure values (142 ± 50 mmHg *versus *99 ± 20 mmHg, p = 0.026), greater delta mean blood pressure values (106 ± 39 mmHg *versus *76 ± 20 mmHg, p = 0.056) and were older (54.7 ± 14.7 *versus *36.2 ± 15.1 years, p = 0.01) than patients with troponin T levels < 0.01 pg/ml. Troponin T was inversely associated with systolic pressure (r = -0.53, p = 0.01) during autonomic instability period.

EKG findings performed at different time points were similar to baseline EKG (including the fact that no new Q waves were identified), except for arrhythmias.

### Transthoracic two-dimensional echocardiography

Transthoracic two-dimensional echocardiography with Doppler was performed at the bedside during autonomic instability. In some patients, important variations of blood pressure occurred during the echocardiograms, but LVEF values remained within normal limits. The exam was repeated in the echocardiography laboratory under optimal conditions, after recovery from tetanus. Mean ejection fraction measured during autonomic instability and in the recovery period was 67 ± 7% and 65 ± 7%, respectively (p = 0.41).

## Discussion

In this study in severe tetanus, all patients had autonomic instability characterized by cardiac arrhythmias, blood pressure instability, and/or cardiac arrest. However, these findings were not associated with increase in plasma catecholamine levels or in biological markers of inflammatory response. In spite of an increase in levels of the myocardial damage marker troponin T in 12 patients, this was not associated with cardiac dysfunction, as assessed by LVEF.

### Autonomic instability and plasma catecholamines

In our study, plasma catecholamine levels were within normal ranges during autonomic instability; in fact, mean levels tended to be higher after recovery from tetanus. Autonomic instability in tetanus suggests intense sympathetic activity [4]; this has been associated with high catecholamine, mainly norepinephrine, levels [9,10,22]. However, some case reports showed contradictory results, reporting elevated [3,23] or normal [6] levels of urinary catecholamine excretion. A case report of one patient, where catecholamines were measured in a hourly basis, showed increase in catecholamine levels during hypertensive periods and normal levels when blood pressure was normal. Urinary levels were normal too [22]. Another study showed increased levels of catecholamines in three patients during periods of hypertension, but the patient who was under curare had near normal catecholamine levels [5]. Differently from our approach, others have measured urinary levels of catecholamines, observing both normal or elevated levels [3,6,23]. From the above studies, measuring either urinary or plasma levels of catecholamines in patients with tetanus, no definite consensus can be determined as to which is the best approach. As during autonomic instability, variation of blood pressure can be very rapid, we chose to measure catecholamines using HPLC, considered a sensitive detection method, in only one moment, which could be temporally associated or not with hypertensive peaks. However, we did not observe increase in catecholamine levels when blood pressure values were at their peak

Normal catecholamine concentrations observed during autonomic instability may also be explained by the pharmacological treatment and resources available nowadays in ICUs, which allow the patient to be kept under deep sedation to control spasms and under adequate mechanical ventilation. Thus, our findings cannot be truly compared to previous reports mentioned above regarding catecholamine levels [3,22,23], mainly because availability of treatment strategies was markedly different. After recovery from tetanus, without anesthesia and under normal conditions, plasma catecholamines may have returned to their usual concentrations; therefore higher than those from the autonomic instability period. Alternatively, other authors postulate that vasomotor disorders in severe tetanus results from changes in systemic vascular resistance secondary to the involvement of the central nervous system [12,13,24]. An alternative would be that autonomic dysfunction in tetanus may not be mediated by plasma catecholamines but neurally, and therefore not reflected by plasma catecholamines levels. In fact, catecholamines activity is complex and involves a multistep G-proteins, protein kinase C, cAMP and phosphodiesterase actions. Coupling between these components appears to be highly modulable [25]. Interestingly, evaluation of autonomic nervous system function with spectral analyses of heart rate variability in a case of tetanus, recently revealed profoundly decreased activity of both sympathetic and parasympathetic modulation of cardiac rhythm, but with predominant parasympathetic nervous system impairment [26].

### TNF-α levels

In this study, levels of TNF-α were not above expected normal limits. To our knowledge, no previous studies attempted to characterize cytokine profile in patients with tetanus, which limits our ability to compare ours to findings from others. Our group, as well as others, has shown that TNF-α levels are increased in the context of sepsis [27]. In this study some patients had documented infection although sepsis criteria were difficult to determine because of autonomic instability. However, patients with tetanus are not fully comparable to patients with sepsis, as tetanus is characterized by an intoxication of the central nervous system by the tetanic toxin, not necessarily an infectious process related/associated with sepsis. An alternative hypothesis was that potentially elevated cytokines in the setting of tetanus could be related to myocardial damage caused by excessive and continuous sympathetic drive [9]. However, in our study no such excessive production of catecholamines was found, further restraining possible sources of increased TNF-α.

### Troponin T levels

In our series, most patients had elevated levels of this marker but no new Q waves were observed in the EKGs performed afterwards. Few reports in experimental *in vitro *models of tetanus have demonstrated increased traditional cardiac enzymes [28]. This is the first time (to our knowledge) that troponins are evaluated in the setting of tetanus; on the other hand, these markers are described to be elevated even in healthy individuals after extraneous physical activity [29], probably indicating some degree muscle damage. Likewise, in our patients it is possible that some degree of myocyte damage occurred but perhaps not clinically relevant to produce noticeable Q waves at the EKG. However, we observed an inverse association of troponin T and systolic pressure, a positive association with diastolic pressure and mean blood pressure variations, suggesting that pressure instability contributed to elevation of troponin T in these patients. Similarly, findings of increased troponins in sepsis have also been found to be associated with duration of hypotension, but not with areas of necrosis by EKG findings [30]. Therefore, our data may suggest that as in SIRS events and in sepsis, blood pressure instability may influence the elevation of troponin concentrations without myocardial necrosis in tetanus.

### Left ventricular ejection fraction

In our study, echocardiography evaluation during autonomic instability failed to identify systolic ventricular dysfunction or regional contractility abnormalities. In spite of the occurrence of variable values of blood pressure during echocardiography, practically all patients maintained normal ejection fraction, indicating a well preserved cardiac reserve in this setting. These findings were confirmed by transthoracic echocardiography performed under ideal conditions after recovery from tetanus. These observations were compatible with the absence of ischemic or necrosis areas by EKG, in spite of elevated troponin T levels.

Previous studies of severe tetanus with autonomic instability suggested the occurrence of myocardial dysfunction secondary to myocardial necrosis caused by tetanic toxin and to elevated catecholamine concentrations [4,8,11,31-33]. A study using invasive hemodynamic assessment showed a profile compatible with hyperdynamic response [14]. Although invasive assessment of cardiac function may not be methodologically comparable to echocardiography-based evaluation, our data, nonetheless, indicate that cardiac function was in fact preserved during autonomic instability, not necessarily reflecting an hyperdynamic pattern, since in both autonomic instability and after tetanus recovery similar/normal values for LVEF were observed.

### Limitations of the study

Only one measurement of catecholamine and TNF-α concentrations was performed during the autonomic instability period, which may not have coincided with the peak of release of these substances. However, according to previous reports available [4,5,22,23] we assumed that significant increases in catecholamine levels were present during the entire autonomic instability period. Serial measurements could have provided different results.

## Conclusion

In patients with severe tetanus, during the period of autonomic instability, our data failed to demonstrate the presence of increased levels of catecholamines or the presence of cardiac dysfunction. Thus, our data do not support the hypothesis that autonomic disturbances in tetanus are secondary to adrenergic variations or cardiac dysfunction. Additionally, it may be suggested that within-normal limits levels of catecholamines in the autonomic instability period may be explained by overblurred of the autonomic system in tetanus.

We speculate that additional mechanisms, perhaps of central origin, may play more important roles in the pathogenesis of autonomic dysfunction in tetanus, and that levels of catecholamines and cardiac dysfunction contribute less importantly. Experimental studies are required to further elucidate cause-effect relationship between these events in tetanus.

## List of abbreviations

APACHE II = Acute Physiology and Chronic Health Evaluation II

ECLIA = electrochemiluminescence immunoassay

EKG = electrocardiogram

HPLC = high performance liquid chromatography

ICU = intensive care units

LVEF = left ventricular ejection fraction

TNF = tumor necrosis factor

## Authors' contributions

JSB participated in study conception, data collection, data analysis and drafting.

NC participated in study conception, study design and coordination, and drafting.

Both authors have read and approved the final version of the manuscript.
